# Value of ultra-high field MRI in patients with suspected focal epilepsy and negative 3 T MRI (EpiUltraStudy): protocol for a prospective, longitudinal therapeutic study

**DOI:** 10.1007/s00234-021-02884-8

**Published:** 2022-01-05

**Authors:** R. H. G. J. van Lanen, C. J. Wiggins, A. J. Colon, W. H. Backes, J. F. A. Jansen, D. Uher, G. S. Drenthen, A. Roebroeck, D. Ivanov, B. A. Poser, M. C. Hoeberigs, S. M. J. van Kuijk, G. Hoogland, K. Rijkers, G. L. Wagner, J. Beckervordersandforth, D. Delev, H. Clusmann, S. Wolking, S. Klinkenberg, R. P. W. Rouhl, P. A. M. Hofman, O. E. M. G. Schijns

**Affiliations:** 1 Department of Neurosurgery, Maastricht University Medical Center, Maastricht, the Netherlands; 2 School for Mental Health and Neuroscience (MHeNs), Maastricht University, Maastricht, the Netherlands; 3 Scannexus, Ultra-High Field MRI Research Center, Maastricht, the Netherlands; 4 Academic Centre for Epileptology, Kempenhaeghe/Maastricht University Medical Center, Heeze/Maastricht, the Netherlands; 5 Department of Radiology, Maastricht University Medical Center, Maastricht, the Netherlands; 6 Department of Electrical Engineering, Eindhoven University of Technology, Eindhoven, the Netherlands; 7 Department of Cognitive Neuroscience, Faculty of Psychology and Neuroscience, Maastricht University, Maastricht, the Netherlands; 8 Department of Clinical Epidemiology and Medical Technology Assessment, Maastricht University Medical Center, Maastricht, the Netherlands; 9 Department of Pathology, Maastricht University Medical Center, Maastricht, the Netherlands; 10 Department of Neurosurgery, RWTH Aachen University Hospital, Aachen, Germany; 11 Department of Epileptology and Neurology, RWTH Aachen University Hospital, Aachen, Germany; 12 Department of Neurology, Maastricht University Medical Center, Maastricht, the Netherlands

**Keywords:** Epilepsy, Epilepsy surgery, UHF MRI, 7 T, 9.4 T

## Abstract

**Purpose:**

Resective epilepsy surgery is a well-established, evidence-based treatment option in patients with drug-resistant focal epilepsy. A major predictive factor of good surgical outcome is visualization and delineation of a potential epileptogenic lesion by MRI. However, frequently, these lesions are subtle and may escape detection by conventional MRI (≤ 3 T).

**Methods:**

We present the EpiUltraStudy protocol to address the hypothesis that application of ultra-high field (UHF) MRI increases the rate of detection of structural lesions and functional brain aberrances in patients with drug-resistant focal epilepsy who are candidates for resective epilepsy surgery. Additionally, therapeutic gain will be addressed, testing whether increased lesion detection and tailored resections result in higher rates of seizure freedom 1 year after epilepsy surgery. Sixty patients enroll the study according to the following inclusion criteria: aged ≥ 12 years, diagnosed with drug-resistant focal epilepsy with a suspected epileptogenic focus, negative conventional 3 T MRI during pre-surgical work-up.

**Results:**

All patients will be evaluated by 7 T MRI; ten patients will undergo an additional 9.4 T MRI exam. Images will be evaluated independently by two neuroradiologists and a neurologist or neurosurgeon. Clinical and UHF MRI will be discussed in the multidisciplinary epilepsy surgery conference. Demographic and epilepsy characteristics, along with postoperative seizure outcome and histopathological evaluation, will be recorded.

**Conclusion:**

This protocol was reviewed and approved by the local Institutional Review Board and complies with the Declaration of Helsinki and principles of Good Clinical Practice. Results will be submitted to international peer-reviewed journals and presented at international conferences.

**Trial registration number:**

www.trialregister.nl: NTR7536.

## Introduction

About 30–40% of patients with epilepsy are drug-resistant, with higher rates for children [1]. Resective epilepsy surgery is a well-established, evidence-based, treatment option for 10–50% of drug-resistant patients, depending on the underlying etiology [2–4]. One of the major predictive factors for good surgical outcome (i.e., seizure freedom) is the detection of a potential epileptogenic lesion/zone on magnetic resonance imaging (MRI) [5–7]. Approximately 30% of adult and pediatric patients with focal epilepsy have no identifiable, potential epileptogenic lesion on MRI, “MRI-negative” [8–10]. Consequently, a considerable number of MRI-negative drug-resistant patients are not eligible for resective surgery, leading to continuation of disease burden and lower quality of life [9].

Recent developments in the field of non-invasive preoperative examinations, like high-density electroencephalography (EEG), EEG-functional MRI (fMRI), single photon emission computed tomography (SPECT), positron emission tomography (PET), magnetoencephalography (MEG), and ultra-high field (UHF) MRI, have improved localization of the epileptogenic zone, leading to surgical treatment in patients who previously were not considered candidates for epilepsy surgery [11–13]. The detection rate of potential epileptogenic lesions on MRI is strongly dependent on technical factors, such as magnetic field strength (in tesla, T), use of phased array head coils, and image (post)processing and analyses [9, 14]. Currently, the clinical state-of-the-art imaging for lesion detection in epilepsy includes a 3-T MRI dedicated epilepsy protocol, reviewed by an experienced neuroradiologist in the field of epilepsy(surgery) [15]. However, even under these optimal circumstances, small structural abnormalities, like focal cortical dysplasias (FCD), can remain unidentified at 3 T [16, 17]. FCDs are congenital malformations of cortical development characterized by aberrant migration and differentiation of neuronal cells [18, 19] and are a major cause of chronic epilepsy, especially in children [20]. It has been reported that FCD is the most frequent lesion (45–51%) in epilepsy surgery candidates who are “MRI-negative” at 3 T [17, 21], and cannot be visualized in up to 40% of the cases [22].

We hypothesize that UHF MRI (7 T and 9.4 T) leads to improved detection and quantification of tiny structural and functional (sub)cortical abnormalities, due to a higher spatial resolution and contrast level beyond what is available at 3 T [23, 24]. By applying 9.4 T, we hypothesize that there will be an additional diagnostic gain compared to 7 T MRI. Additionally, this increased lesion detection of UHF MRI, and additional voxel-based morphometry postprocessing techniques of the UHF images, will guide surgical decision-making, leading to a more personalized type of epilepsy surgery and improved postoperative seizure outcome, with a higher chance of becoming seizure free in potential candidates for epilepsy surgery.

The primary objectives of this study are (1) to estimate the detection rate of structural and functional brain lesions on 7 T and 9.4 T MRI in patients with drug-resistant 3 T MRI-negative focal epilepsy and (2) to assess whether this increased detection rate is associated with improved postoperative seizure outcome. In this paper, we present the study protocol according to Standard Protocol Items: Recommendations for Interventional Trials protocol guidelines [25].

## Methods and analysis

### Study setting and population

**Fig. 1:**
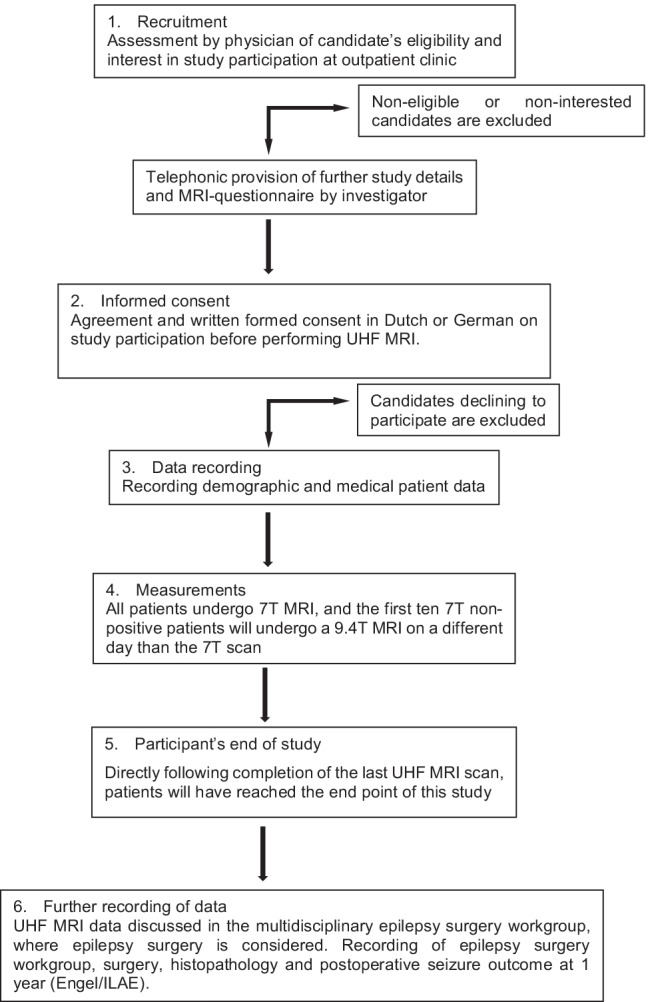
Study participants’ timeline

This is a multicenter prospective, longitudinal, therapeutic study assessing the value of UHF MRI for lesion detection and therapeutic impact of this improved lesion detection, in patients with a suspected focal cause of drug-resistant epilepsy and unrevealing clinical 3 T MRI, who are candidates for resective epilepsy surgery (Fig. 1).

The diagnosis of focal epilepsy and a suspected region of interest (ROI) as the focal epileptogenic zone is based on thorough examinations including medical history with seizure semiology, a video-EEG, neuropsychological examination, and 3 T MRI according to a dedicated epilepsy protocol [15, 26], including morphometric analysis program (MAP18) postprocessing [27, 28]. In selected cases, a PET scan and/or subtraction ictal SPECT co-registered to MRI (SISCOM) and/or EEG triggered fMRI and/or MEG are performed. In a subset of these patients, with discordant results from non-invasive examinations, intracranial EEG recordings by implanted surface or intracerebral depth electrodes are necessary. Subsequently, a multidisciplinary team, consisting of neurosurgeons, neurologists, clinical neurophysiologists, neuropsychologists, and neuroradiologists, assesses eligibility for epilepsy surgery. All clinical 3 T MRI scans are reviewed by a radiologist specialized in epilepsy imaging, and are considered “negative” only after review of the scan in the multidisciplinary team, where the 3 T scan and other non-invasive examination are considered.

### Inclusion and exclusion criteria

For the present study, we include pediatric patients (children, aged 12–17 years) and adults (aged 18 and above), with drug-resistant focal epilepsy (ongoing seizures despite at least two adequate and well-tolerated anti-epileptic drugs trials [29]), who are under evaluation for resective epilepsy surgery. All patients have had a clinical 3 T MRI without an identified explanatory epileptogenic lesion. Exclusion criteria are intellectual disability (IQ < 70), incapacity to sign informed consent, pregnancy, and criteria specific for UHF MRI (see Table 1).

**Table 1:** Overview of inclusion and exclusion criteria

Inclusion criteria
Age ≥ 12 years
Drug-resistant focal epilepsy
Work-up for epilepsy surgery
Clear suspicion on the focal onset of the epilepsy
Absent explanatory abnormalities on conventional 3 T MRI
Informed consent signed
Exclusion criteria
Incapacitated to sign informed consent
Patients and/or legal representative have an intellectual disability (IQ < 70)
Pregnancy
MRI-exclusion criteria:
Claustrophobia
Pacemaker, neurostimulor, insulin pump or other pump
Aneurysm clips in cerebro not safe at 7 or 9.4 T MRI
Metal particles in the head (incl. eye)
Hearing prostheses (not all types)
Tattoos above the diaphragm
Other body implants not proven safe at 7 or 9.4 T MRI
Relative contra-indications depending on place and kind
Artificial heart valves
Joint prostheses
Obesity making MRI-scanning impossible due to size

### MRI-protocol

MRI acquisition will be performed at Scannexus BV, a specialized UHF MRI research facility, using an actively shielded Siemens Magnetom “classic” 7 T with whole-body gradients and 32-channel NOVA head coil, and a Siemens Magnetom 9.4 T with head-gradient set and 31-channel receive/16-channel parallel RF transmit head coil.

**Fig. 2:**
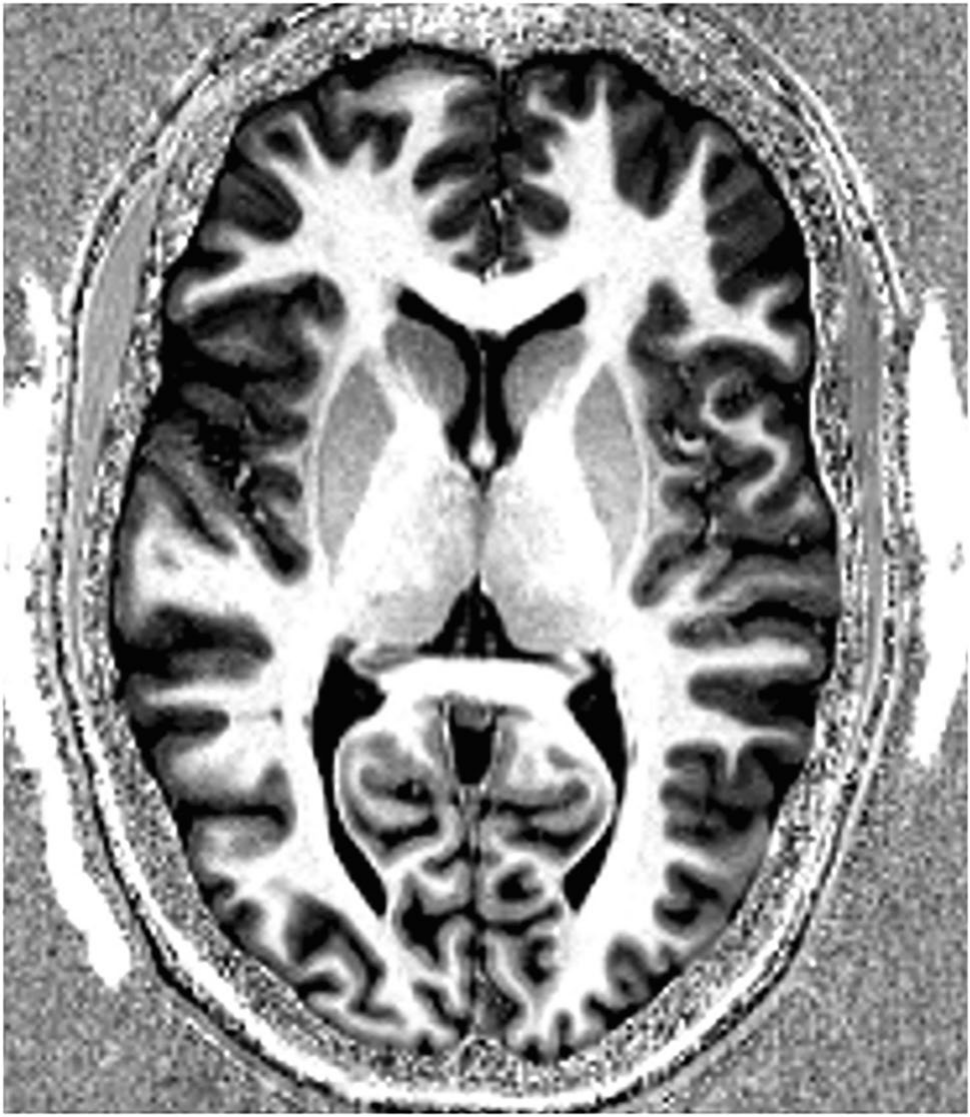
7 T 3D T1-MP2RAGE axial image of a test subject’s cerebrum with an isotropic voxel size of 0.7 mm. The di-electric pads can be seen on both sides

**Fig. 3:**
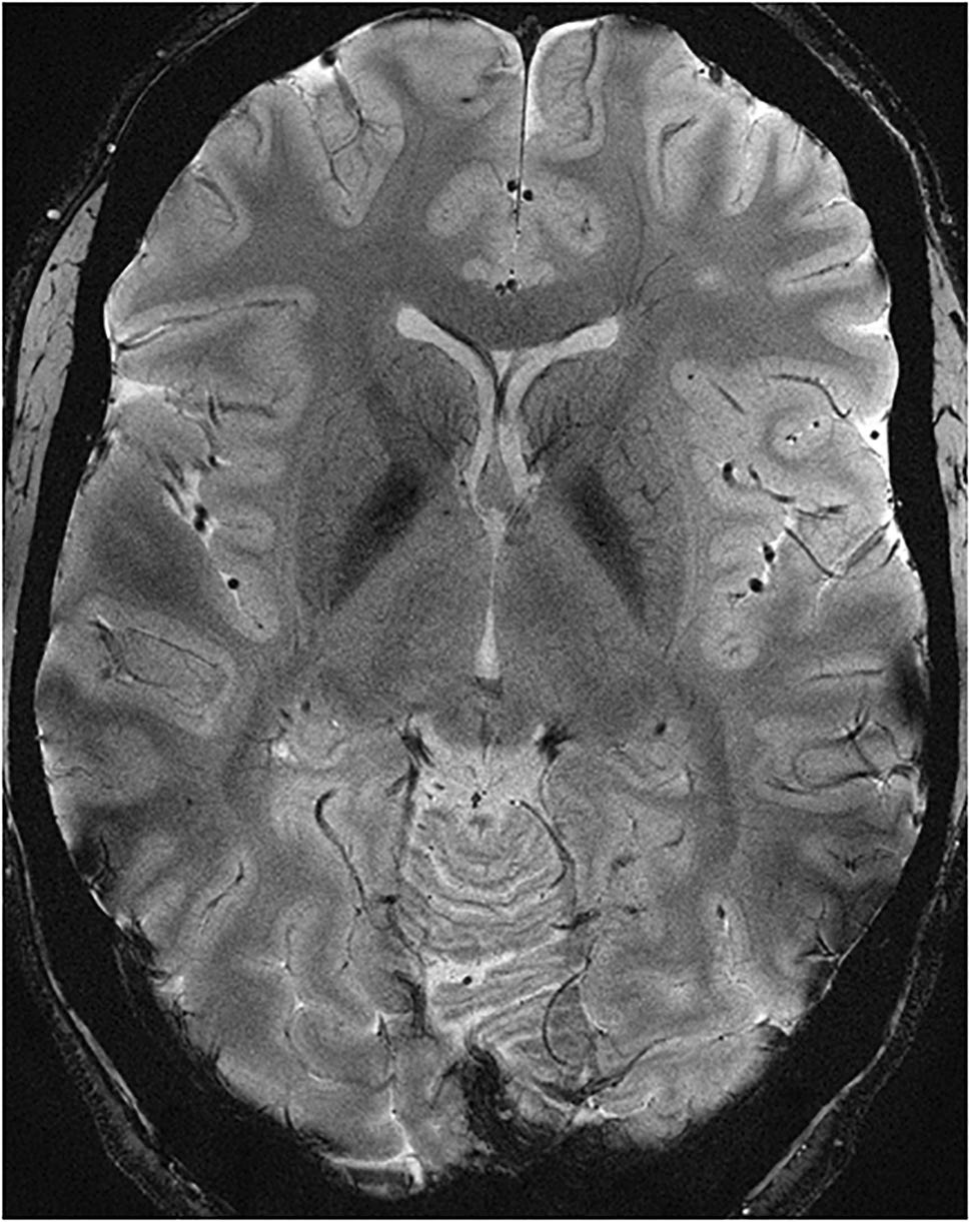
7 T 2D dual-echo GRE (T2*) axial image of a test subject’s cerebrum with a voxel size of 0.3 × 0.3 × 2.0 mm

Development of a clinically applicable 7 T and 9.4 T MRI scan protocol for detection of structural abnormalities is part of this study. We created a format for a proposed UHF scan protocol (see Table 2). All sequences will acquire images of the whole brain. This scan protocol allows for the use of normative control data, acquired by Haast et al. [30]. Two dielectric CaTiO_3_ pads with demineralized water are used to improve the inhomogeneous MRI signal in the temporal lobes. As a preparation, we scanned some controls at 7 T to assess image quality (see Figs. 2 and 3).

**Table 2:** Proposed UHF MRI scan protocol, consisting of 3D T1-MP2RAGE (magnetization prepared two rapid gradient echoes), 3D SPACE FLAIR (fluid-attenuated inversion recovery), 2D T2-SPACE, 2D dual-echo GRE (spoiled-gradient echo), 3D GRE ASPIRE, BOLD (blood-oxygen-level-dependent) resting-stage functional MRI, DTI (diffusion tensor imaging), ASL FAIR (arterial spin labeling flow-sensitive alternating inversion recovery), and total acquisition time ± 70 min

	Type	Sequence	Aimed voxel size (mm)
-	Localizer		1.1 × 1.0 × 3.0
-	B0 map		2.9 × 2.9 × 4.0
-	B1 map		3.9 × 3.9 × 5.0
1	Structural	3D T1-MP2RAGE	0.7 × 0.7 × 0.7
2		3D FLAIR	0.8 × 0.8 × 0.8
3		2D T2-SPACE	0.6 × 0.6 × 2.0
4		2D dual-echo GRE	0.3 × 0.3 × 2.0
5		3D GRE ASPIRE	0.7 × 0.7 × 0.7
6	Functional BOLD	BOLD rs-fMRI	1.4 × 1.4 × 1.4
7	Diffusion	DTI	1.05 × 1.05 × 1.05
8	Functional ASL	ASL FAIR	2.8 × 2.8 × 2.8

### Intervention and evaluation protocol

Patients are included after obtaining written informed consent; subsequently, MRI will be performed. In a first run, both 3 T and UHF images will be independently evaluated by two neuroradiologists experienced in epilepsy-imaging, and a neurosurgeon or neurologist specialized in epilepsy surgery, without prior knowledge of previously obtained non-invasive examinations and the potential epileptogenic zone (= ROI). In a second run, first the 3 T, subsequently, the UHF MRI scans will be evaluated with prior knowledge of the ROI by the same investigators. The images will be reviewed for image quality and potential epileptogenic lesions using a structured UHF MRI scoring form, allowing objective comparison between observers and scans. Lesion size/volume, number, lateralization, localization, shape, and signal intensity detected on the different sequences are marked. The mesiotemporal structures (hippocampus, amygdala) are evaluated for abnormalities and asymmetries in volume and signal intensity. Evaluation for FCDs is done by screening for features of blurring cortical-subcortical junction/cortical thickening/transmantle sign/hyperintensity of grey and white matter/abnormal gyral-sulcal pattern/segmental or lobar hypoplasia/hypoplasia with loss of regional white matter volume. Additionally, we evaluate features of an intracortical black line as a possible marker of a FCD and magnetic susceptibility effects due to tissue irregularities [31]. Additionally, susceptibility effects are used to detect the presence of abnormal vasculature adjacent to areas of cortical dysplasia or polymicrogyria [32]. A diffusion sequence (by acquiring diffusion tensor imaging, DTI) is firstly performed to aid the detection of intra-cortical radial and tangential diffusion abnormalities, suggestive for FCD types 1a and 1b, and secondly to detect a difference in intra-cortical radial and tangential diffusion between FCD types 1a and 1b. Using post-processing, additional FA and ADC maps will be generated. Abnormalities in blood-oxygenation level fluctuations are detected using resting-state fMRI and arterial spin labeling (ASL). Furthermore, we will make use of voxel-based morphometry (VBM) structural post-processing technique. The aim of this technique is to quantitatively analyze morphology and signal intensity on both 3 T and UHF scans by using algorithms for automated FCD detection as qualitative visual inspection of the MRI will fail to detect tiny aberrances [33].

Results of the UHF MRI scans will be presented and discussed in a monthly multidisciplinary epilepsy surgery conference. We will evaluate the association between the presence and absence of an abnormal finding, with information of other non-invasive examinations. Additionally, we will assess the potential improvement in detection rate after adding prior knowledge of the ROI to the results of reviewing, by comparing the first with the second run, along with intra-observer and inter-observer variability.

After obtaining UHF MRI results, patients can undergo either (1) resective epilepsy surgery, (2) implantation of intracerebral depth electrodes for intracranial EEG recordings (S-EEG), (3) S-EEG guided radiofrequency thermo coagulation (RFTC), or (4) proceed to palliative treatment options (e.g., vagus nerve stimulation). Surgical resection is the first choice due to its efficacy and will be performed when possible, with regard to lesion location, extent, and expected lesion etiology. Depth electrode implantation will be used when additional information on the possible epileptogenic zone is required. S-EEG RFTC will be performed when resective surgery is not recommended, for example, in case of deep-seated periventricular heterotopias. Palliative treatment options are explored when other curative treatments are excluded. Samples of surgically treated patients will be examined by routine histopathological analysis and classified according to ILAE guidelines [20]. In case of resective epilepsy surgery, UHF MRI–based diagnosis will be compared to histopathological diagnosis. Postoperative seizure outcome following ILAE and Engel classification [34] will be assessed by the treating neurologist as standard care minimally 1 year after surgery.

### Outcome parameters

The primary outcome parameter of this study is the number of patients with detected “epileptogenic” structural and functional brain abnormalities on 7 T and 9.4 T UHF neuroimaging, the so-called “diagnostic gain” in patients with 3 T MRI-negative drug-resistant focal epilepsy. The second primary outcome parameter is postoperative seizure outcome (ILAE/Engel class at 12 months) in operated UHF MRI-positive versus MRI-negative patients “i.e., therapeutic gain.”

As secondary outcome parameters, detection rate of UHF resting-state fMRI for cortical abnormalities in spontaneous blood oxygenation level-dependent (BOLD) signal fluctuations as a marker of an epileptogenic lesion, concordance of UHF MRI imaging with histopathological diagnosis of surgical specimen, intra- and inter-observer agreement for UHF MRI evaluation, and association between ROIs in the non- or semi-invasive work-up modalities and UHF MRI data will be assessed.

### Recruitment capacity, consent, and timeline

Recruitment of eligible study participants will take place at (1) both locations of the Academic Center for Epileptology (ACE) Kempenhaeghe/Maastricht University Medical Center + (MUMC +), Heeze/Maastricht, The Netherlands, and (2) the RWTH Aachen University Hospital, Section of Epileptology and Department of Neurosurgery Aachen, Germany. Based on feasibility and number of patients, we expect 30 patients from Kempenhaeghe/MUMC + , among which eight children 12 to 18 years, to qualify for this study annually. Recruitment from Aachen is expected to be 10 patients annually. Taking into account, a probable informed consent rate of 75% results in an annual inclusion of 30 patients.

The study participants’ timeline is outlined below; an overview is also given in Fig. 1.

1. Recruitment
  All patients ≥ 12 years discussed in the epilepsy surgery workgroup will be analyzed for study eligibility. The treating physician will inform the patient about the main study characteristics, asked whether they, and in case of children the legal representative, is interested in more information and agrees to be contacted by phone by the investigator. Upon approval, the patient information letter (PIF) and informed consent form will be provided to the subjects. A minimum reflection time of 1 week is taken into account. Participants can contact the local investigator digitally. If no reply is received within 3 weeks, the investigator will contact the patient by phone. Patients will be informed that they can decide to end their participation at any time.
2. Informed consent
  Before performing the UHF MRI scan, a Dutch or German informed consent agreement form will be signed in duplicate for Dutch or German participants, respectively.
3. Data recording
  Following informed consent agreement, patient data will be recorded from digital patient files. Digital patient files encompass the electronic patient dossier (EPD) at the ACE MUMC + , ACE Kempenhaeghe, and RWTH Aachen University Hospital, containing the patient files from the multidisciplinary epilepsy surgery workgroup. MRI risk questionnaires will be filled out by patients and controlled by the MRI technician before performing MRI.
4. Measurements
  All patients will undergo 7 T MRI, and the first ten 7 T MRI-negative patients will undergo 9.4 T MRI on a different day than the 7 T scan, as described in the intervention protocol section.
5. End of study for the participant
  Directly following completion of the last UHF MRI scan, patients will have reached the end point of this study.
6. Further recording of information

Data obtained by UHF MRI (part 4. Measurements) will be discussed in the multidisciplinary epilepsy surgery workgroup and, when consensus is reached about possible epileptogenicity of a novel lesion, stereo-EEG, respective, or minimally invasive surgery will be considered.

Results from the epilepsy surgery workgroup, surgery, histopathology, and postoperative seizure outcome at 1 year will be recorded from digital patient files. If patients do not undergo epilepsy surgery, conclusions, and recommendations from the workgroup, next to possible other treatment suggestions and seizure follow-up will be recorded.

### Sample size

The primary study outcome is the lesion detection rate of UHF MRI. This outcome will be estimated without formal null-hypothesis testing. We previously conducted a study in which we included 19 adult patients where MEG directed to an epileptogenic lesion [35]. All patients were 3 T negative; on 7 T, six patients showed possible epileptogenic lesions. However, as there is no similar research based on other examination modalities (SPECT, PET, etc.) available for UHF MRI imaging, no data to perform a sample size calculation is available [23, 24]. Therefore, we take a pragmatic approach to the determination of sample size. Rules of thumb for linear regression analysis state that at least 10 observations are needed for each variable that is entered in the regression model. In addition to univariate analysis, we aim to perform multivariable analysis in which we will correct for up to five potential confounding variables. Hence, we will need to include at least 60 patients for this study. With a sample of this size, the margin of error (i.e., half-width of the 95% confidence interval) of the detection rate will likely be below 0.20.

### Data processing

#### Procedures

This study complies with the Declaration of Helsinki and will be conducted in accordance with the principles of Good Clinical Practice (GCP). Standardized processing files for obtaining informed consent, measurement procedures, reporting (serious) adverse events (AEs), and recording patient and measurement data parameters in electronic case report file (eCRF) are available. Investigators obtaining patient informed consent, conducting UHF MRI scans, and recording eCRF data will have received specific training beforehand.

#### Data management

Patients’ demographic and clinical data are recorded in an eCRF at a secure encrypted database (Castor Electronic Data Capture), which enables an audit trail and is GCP certified. Reconstructed MRI data of all patients will be stored in the Radiology Research Server of ACE MUMC + , and outcome is recorded anonymized in the eCRF. After verification of recorded data to source data by one of the executive investigators, recorded data in the eCRF by Castor EDC will be exported to an SPSS file for further statistical analysis.

Patients will be assigned a numeric sequential study number to identify all clinical data, documented in a separate screening database held on a secure computer at the including study site. Source data, code encrypting document, and coded data in the study database are locked and only accessible to the principal and executive researchers and monitors. In each patient file, we will note coded information under which results are traceable, ensuring that the epilepsy surgery workgroup can have access to all possible useful information. Additionally, the “Inspectie Gezondheidszorg en Jeugd” (healthcare and youth inspection) will be provided with the possibility to review research data whenever this is required. On completion of the study, the study database will be locked and data are securely archived for 15 years in accordance with local policy.

### Safety

The clinical trial sponsor (MUMC +) and principal investigators (OEMGS and AJC) have overall responsibility for the execution of this study including safety. Individual investigators will be responsible for reporting AEs and serious AEs (SAEs) to the principal investigators. SAEs are defined as AEs resulting in death, life-threatening events, prolonged hospital stays, or significant disability. Up till now, there are no reported (S)AEs associated with the clinical use of UHF MRI in literature. UHF MRI is, with regard to safety, highly comparable to standard MRI scanning at 3 T for clinical health care when safety precautions are taken into account [36, 37]. The MRI safety relevant inclusion/exclusion criteria for 7 T and 9.4 T are set by the local Safety Review Board (SRB), which acts as an expert advisory panel on behalf of the local Medical Ethical Committee/Institutional Review Board (IRB). The MRI-questionnaire determines whether a patient is eligible and safe to undergo the scan.

Claustrophobia can be a possible concern of UHF MRI, due to a longer magnet bore. However, this is accounted for by excluding patients with known claustrophobia. Also, all subjects already underwent a clinical 3 T MRI scan; any occurrence of adverse events during this scan would lead to exclusion of the subject. One important frequently reported side-effect at UHF is vertigo when moving in/out the bore, in some cases resulting in nausea or small involuntary eye motion, i.e., nystagmus [38]. However, this poses no health threat to patients [36]. In case patients experience uncontrollable nausea, scanning will be stopped immediately. All events or complaints from signing informed consent until 1 day after completion of the UHF MRI scan will be considered an adverse event, and are reviewed by the principal investigator to decide if there is a causal link and, when applicable, appropriate action will be undertaken. Follow-up in patients will continue until minimally 1 year after epilepsy surgery. Due to the nature of the investigated pathology (epilepsy), seizures and subsequent sequelae between completion of the UHF MRI scan and 1-year follow-up will not be considered an adverse event related to study procedures. SAEs will be reported through the web portal to ToetsingOnline to the IRB (METC azM/UM). Liability and subject study-related insurance are provided.

### Statistical analysis

Baseline characteristics of the cohort will be described in detail as mean and standard deviation or median and interquartile range for continuous variables (depending on their normality of distribution) and as count and percentage for categorical variables. Missing values are unlikely to occur, but in case of incomplete MRI data, we will use stochastic regression imputation to facilitate the inclusion of all study participants in the analyses.

UHF MRI data, such as characteristics of and, if applicable, localization of abnormalities, will be summarized using descriptive statistics. Primary outcomes diagnostic and therapeutic gain will be expressed as point estimates with 95% confidence intervals. Explorative analysis is used for quantitative MRI measurements (diffusion, fMRI, ASL). Radiological assessment will be done independently, in case of different results consensus will be sought. Intra-observer and inter-observer agreement in assessing MRI images will be quantified using Cohen’s kappa for categorical MRI parameters, and intra-class correlation coefficient (ICC) for continuous MRI parameters. Associations between qualitative findings and histopathological abnormalities will be computed using Spearman rank correlation, point bi-serial correlation, or Fisher’s Exact test, depending on the distribution of the findings.

We will assess the association between demographic, clinical parameters, and outcome measures by using univariable and multivariable regression analysis. A *p*-value of < 0.05 will be considered statistically significant. Statistical analysis will be performed using IBM SPSS software version 25 or higher.

### Monitoring and auditing

This study was classified as intermediate risk by the local data monitoring committee (Clinical Trial Center Maastricht (CTCM)). Monitoring visits by the CTCM include a review of consent and study procedures according to study protocols, source data and audit trail verification, and the review of (serious) AE reporting. Monitoring is independent and performed at least once a year and can be unannounced. Monitor evaluations are reported to the local IRB.

### Ethics and dissemination

This research protocol has been approved by the local IRB (Medisch-ethische toetsingscommissie academisch ziekenhuis Maastricht/Maastricht University (METC azM/UM)) and has been assigned the following protocol ID: NL66929.068.18. Approval of the IRB of Kempenhaeghe Epilepsy Center (ID METC KH 19.14) and the RWTH Aachen University medical faculty was gained (CTC-A 20–302). This study has also been registered at the Netherlands National Trial Register (ID: NTR7536), which is acknowledged by the WHO and International Committee of Medical Journal Editors (ICMJE). Results will be recorded using audit trails to increase reproducibility. Study protocol and results will be submitted to peer-reviewed journals and presented at international conferences.

## Discussion

Patients with drug-resistant focal epilepsy, who are MRI-negative, are less likely to be considered candidates for surgery compared to MRI-positive patients and have less satisfactory seizure outcome after surgery [17, 39]. MRI-negative patients who do undergo surgery frequently have distinct epileptogenic lesions identified post-surgery via histopathological investigations or retrospective examination of the images [40, 41]. Therefore, it seems justified to conclude that improving detection rate for epileptogenic lesions improves postoperative seizure outcome.

### Diagnostic value

The added diagnostic value of UHF, compared to lower field strengths, has been demonstrated for distinct pathologies, such as vascular malformations [42], hippocampal sclerosis [43], brain tumors [44], and degenerative brain diseases [45]. Over the last years, some studies demonstrating the diagnostic gain of 7 T MRI over clinical 1.5 T or 3 T MRI in patients with drug-resistant focal epilepsy have been published [23, 24, 46–48]. We recently published a systematic review discussing UHF MRI in human epilepsy [49].

UHF MRI offers a higher signal/contrast-to-noise ratio, enabling higher spatial resolution with better depiction of micro-anatomical structures, therefore improving detection rate of epileptogenic lesions [50]. Studies comparing diagnostic yield of 3 T versus 1.5 T MRI in patients with focal epilepsy have shown this [9, 51]. An additional factor contributing to the diagnostic gain might derive from the use of a dedicated protocol. Interestingly, very recently, a 7-T epilepsy task force published a consensus paper with recommendations on the use of 7 T in clinical practice, based on multicenter and multinational experience with 7 T [52]. The proposed scan protocol for this study includes the considered “minimum scan requirements” and recommendations of this task force, with the addition of what we consider promising sequences, also based on findings of our systematic literature review [15, 49, 52]. Due to the high spatial resolution and sensitivity to the magnetic susceptibility properties of tissues, GRE (T2*) imaging allows better evaluation of the different components of the cortex [52]. Furthermore, GRE contrast increases with increasing magnetic field, hence the particular interest of its value in this study. FLAIR imaging, emphasizing signal changes of the cortex and along the cortical-white matter interface, and at 7 T might uncover even slight signal hyperintensities not otherwise visible [49, 52]. Several MRI features characterize FCD, but with great variety in conspicuity [53]. Especially in mild malformations of cortical development and FCD type I, changes can be subtle and indistinguishable from signal averaging and partial volume effects [54]. Very high isotropic resolution diffusion sequence (close to 1 mm isotropic) achievable in the whole human brain at UHF[55] has been shown to be sensitive to the intra-cortical radial and tangential diffusion direction [56]. This may enhance detectability of FCD type I, and differential abnormalities in radial and tangential diffusion could help stratification into FCD type Ia and type Ib.

Besides structural imaging, growing evidence demonstrates that connectomics, such as functional connectivity, could serve as a marker for pathological functions and networks, especially in a network-disease like epilepsy [57]. Other studies have shown early results that functional connectivity is homotopically correlated with resting-state default-mode networks, visualized by resting-state fMRI [58]. These imaging techniques can couple functional abnormalities to structural lesions, which has been demonstrated in a study by Gupta et al., where abnormalities in spontaneous blood oxygenation level-dependent fluctuations in the perilesional area of FCDs were found [59]. Another recent study confirmed this interesting new direction [60], while functional ASL allows additional measurement of baseline cerebral blood flow [61]. It has been shown that vascular abnormalities are associated with the underlying dysplastic cortex and even (pre)ictal neurovascular and metabolic coupling surrounding a seizure focus [32, 46, 62]. Besides further enhancing diffusion and susceptibility imaging at UHF, abnormalities in BOLD fMRI has been shown to correlate with epileptic foci, especially FCD [59, 63, 64]. Inclusion of these “non-structural” sequences in our scan protocol aims at exploring the additional value of these sequences.

The next step to improve diagnostic yield is the use of a structural post-processing technique, voxel-based morphometry, with its own specific diagnostic properties [65, 66]. The add-on application of 9.4 T MRI in the EpiUltraStudy is intended to provide the next step in examining the possible role of 9.4 T MRI for lesion detection in MRI-negative patients with epilepsy. Since previous 7 T studies have shown a ± 30% increase in detection rate [23, 24, 47], applying 9.4 T MRI could further improve signal-to-noise by up to a factor of two over 7 T[67], and should lead to an additional increase in detection rate.

However, improving imaging only by using more modern MRI-equipment might not be sufficient. An “a priori” hypothesis on the probable location of the epileptogenic zone is crucial to zoom in around a predefined ROI in order to increase detection rate of subtle and potential epileptogenic lesions [11, 12]. This a priori hypothesis can be formulated by application of a variety of non-invasive examinations, for example source localization by pre-surgical MEG-guided 7 T MRI analysis, which results in a clear gain in number of detected abnormalities [35]. PET, SPECT, and EEG-fMRI are other non-invasive modalities and are applied next to clinical semiology, video-EEG, and neuropsychological tests in the pre-surgical work-up of epilepsy surgery candidates to determine a plausible lateralization and/or localization of the epileptogenic focus. If the results of the aforementioned modalities lead to a robust hypothesis of the seizure-onset zone, a ROI can be defined to be examined using UHF MRI.

### Therapeutic value

The EpiUltraStudy will contribute to improve preoperative counseling of the patient with significant and direct benefits for epilepsy surgical candidates. The epileptogenic character of novel detected abnormalities needs to be confirmed in selected cases, after which these patients will be offered epilepsy surgery. On the one hand, it enables the neurologist and neurosurgeon to inform patients on the possible cause of their epilepsy. On the other hand, inclusion of UHF MRI in preoperative workup will lead to a more targeted operation planning, a higher chance of complete lesionectomy, and potentially a minimally invasive surgical treatment. Altogether, the acquired information will lead to an increased understanding of patho(physio)logy in epilepsy and probably will improve the quality of life of these patients. Especially in children, the achievement of seizure freedom, and consequently tapering of the anti-epileptic drugs, leads to improvements in different domains like cognitive function, development, and quality of life [68, 69]. Besides, as children pose the largest group for congenital abnormalities leading to epilepsy, it is expected that the largest part of structural abnormalities, detected on UHF MRI, will be found in children and patients with childhood onset of their epilepsy.

### Challenges

Sample size calculation for a study assessing diagnostic gain and improvement of postoperative seizure outcome is not trivial as there is no comparable research based on other modalities (SPECT, PET, etc.). Thus, no power calculation based on prior studies can be performed. Therefore, we took a pragmatic approach with sample size calculation based on observations needed for each variable entered in a regression model. Final study power has to be established.

The main technical challenges for UHF MRI acquisition are inhomogeneities in the main magnetic field (*B*_0_) and radiofrequency transmit field (*B*_1_) and their stronger interaction with any implants. The current proposed scan protocol might lead to increased sensitivity to motion artifacts due to longer scanning times in the chosen highest resolution protocols, as the patient has to lie still for a longer period of time. Our total scan time of ± 70 min is slightly above acquisition times of other 7 T studies, where acquisition time was not an issue [23, 31, 35, 47]. Additionally, clinical 3 T MRI is performed at an earlier stage than UHF MRI in the epilepsy surgery workup. The results of additional examinations possibly lead to novel insights on the ROI, influencing reviewing of UHF MRI scans. A potential bias may occur because not all patients receive all other non-invasive examinations, but at the clinician’s discretion. Furthermore, inexperience in clinical reading/interpretation of 7 T and 9.4 T images can be an issue, as reviewers might experience a reviewing learning curve. Also, UHF might produce artifacts or geometric disturbances not seen on conventional MRI, potentially resulting in detection of non-relevant lesions (false positives). To address this issue, several UHF pilot scans will be performed to gain experience at reviewing UHF MRI. This includes patients with positive 3 T MRI for a small lesion, along with non-epileptic volunteers.

#### Conclusion

The ultimate aim of the EpiUltraStudy is to examine the added diagnostic and therapeutic value of UHF MRI for lesion detection in patients with drug-resistant focal epilepsy of suspected origin and negative 3 T MRI. Consequently, we hope to improve surgical outcomes by increasing the detection rate of subtle lesions like FCD or initial stage hippocampal sclerosis in patients with 3 T MRI-negative, drug-resistant focal epilepsy and by tailoring the resection into a personalized surgical procedure. Ultimately, UHF MRI imaging could be implemented into standard pre-surgical workup for epilepsy surgery.

The add-on application of 9.4 T MRI is to further investigate the additional benefit of UHF for lesion detection and radiological diagnosis. The EpiUltraStudy is intended to provide the next step in examining the possible role of 9.4 T MRI for lesion detection in 7 T MRI-negative patients with epilepsy. Since previous 7 T studies have shown a ± 30% increase in detection rate [23, 24, 47], applying 9.4 T MRI could further improve signal-to-noise by up to a factor of two over 7 T [67]. This should lead to an additional increase in detection rate, and will further increase the likelihood of candidates for epilepsy surgery to become seizure free.
